# Epidemiology of Peripheral Arterial Disease in Women

**DOI:** 10.2188/jea.13.1

**Published:** 2007-11-30

**Authors:** John P. Higgins, Johanna A. Higgins

**Affiliations:** 1 Department of Medicine, University of Oklahoma.; 2 Department of Medicine, University of Oklahoma, Tulsa.

**Keywords:** peripheral arterial disease, women, epidemiology

## Abstract

BACKGROUND: Peripheral arterial disease is a common disease, which increases with age and presence of vascular risk factors. The extended longevity in industrialized nations coupled with the expanding elderly female population is predicted to lead to an increase in the prevalence of this condition. Little attention has been focussed on gender differences in peripheral arterial disease, or its epidemiology in women.

METHODS: MEDLINE search of English-language reports published between 1966 and 2002 and search of references of relevant papers.

RESULTS: Across various populations of women with different ages and risk factor levels, the prevalence of peripheral arterial disease ranged between 3% and 29%. Diagnosis in women using a sensitive and specific non-invasive test, the ankle-brachial index, detects about 3-5 times the cases than those diagnosed by history of intermittent claudication alone. Contrary to earlier beliefs, prevalence of peripheral arterial disease is similar in women and men, and women may have more asymptomatic disease. Importantly, women with peripheral arterial disease have 2-4 fold increases in cardiovascular morbidity and mortality. Risk factors for peripheral arterial disease appear to be similar in men and women, however relative risks vary somewhat.

CONCLUSIONS: Performing non-invasive testing (e.g. ankle-brachial index) can better diagnose peripheral arterial disease in women than history alone. These women share risk factors with other vascular diseases (coronary and cerebrovascular disease) and should undergo risk factor modification to reduce their cardiovascular morbidity and mortality. More research is needed including aggressive risk factor management in women with asymptomatic peripheral arterial disease.

Peripheral arterial disease (PAD) is a disease whose risk increases with age, and raises one’s risk of death between two and six fold over a ten year period.^1^^,^^2^ Extended longevity in industrialized nations has resulted in the 80 and over population being the most rapidly growing age group.^3^ Many countries currently have at least 10% of their populations age 65 years and older, with expected dramatic growth in this percentage by the year 2020.^4^ Additionally, projections predict that up to two-thirds of those 65 years and older will be female.^5^ An increase in PAD prevalence may occur if the population continues to age without significant alterations in risk factor levels. Indeed, women may represent the majority of PAD patients in the coming decades.

The influence of gender on PAD has gained little attention in the literature; certain studies treat men and woman collectively prior to their statistical analysis. Some report gender differences, others do not. Between 3 to 29% of women aged 45 to 93 years have evidence of the disease when sensitive and specific non-invasive testing is used as a diagnostic tool.^6^^-^^10^ There is considerable evidence that the number of PAD cases currently recognized by physicians, mainly those with symptoms, e.g. intermittent claudication, represents only a minority, referred to by some authors as “the tip of the iceberg.”^6^^,^^7^^,^^11^^,^^12^ The incidence and prevalence of intermittent claudication increases with age, and in most studies, are greater in men than in women. Women may present as many if not more cases than men when asymptomatic PAD prevalence is determined,^7^^,^^8^^,^^13^^,^^14^ usually by ankle-brachial index (ABI).

Risk factors for PAD in women are similar to those for coronary and cerebrovascular arterial disease. Cigarette smoking, total cholesterol, and low-density lipoprotein cholesterol are associated with a higher relative risk for PAD in women than in men in some studies;^10^^,^^15^ other studies find the risks equivalent for women and men.^16^ Importantly, cigarette smoking continues to rise among women in many developed countries, and thus they induce higher risks for PAD. The remainder of the risk factors studied (elevated systolic blood pressure, diabetes mellitus, diminished high-density lipoprotein cholesterol, elevated triglycerides, fibrinogen and homocysteine) afford an equivalent risk for PAD in both sexes.

Due to overlap of these risk factors with other vascular diseases, many women with PAD are also at risk for myocardial infarction and stroke. PAD is also a strong and independent marker for subsequent all-cause mortality in women.

To better identify and manage these high-risk female PAD patients, screening in the form of peripheral pulse examination as well as ABI evaluation could be used. In addition, PAD and its vascular risk factors are under treated in both pre- and post-menopausal women.^6^

The epidemiology and significance of PAD in women is the topic of this review.

## MATERIALS AND METHODS

### Literature Search

In preparation for this paper, a review of the English-language scientific literature was conducted. This was performed primarily by searching the MEDLINE databases for the time period 1966 through 2002. Keywords used in the search included “peripheral,” “arterial,” “disease,” “vascular,” and “women” or “female.” In addition,the medline search was performed using the MESH heading “Peripheral Vascular Diseases” in order to capture all PAD literature that included studies of both sexes. The bibliographies of articles found through the MEDLINE search were also searched for relevant articles, as well as current journal review papers. After removal of papers dealing with carotid, coronary, or congenital vascular disease, as well as case reports, 310 articles remained. This included a total of 72 articles principally relating to diagnosis, 21 to epidemiology, 45 to gender differences, 73 relating to risk factors; the remainder related to management.

### Definition of PAD

PAD is atherosclerosis of the lower limb arteries below the bifurcation of the abdominal aorta.^7^ This commonly manifests as a cramping pain in the large muscle groups of the lower extremities which occurs while walking and relieved with rest, referred to as intermittent claudication.^15^ However, it is important to note that not all claudicants have PAD; nor do all PAD patients develop claudication. The majority (50 to 82%) of PAD patients do not have claudication.^7^^,^^9^^,^^10^^,^^17^

In the International Statistical Classification of Diseases and Health Related Problems 10th revision, PAD is coded under ICD-170.2 “Atherosclerosis of arteries of extremities” under General heading 170 “Atherosclerosis” within Chapter IX “Diseases of the Circulatory System.”^18^ Regarding anatomical distribution, autopsies at Oxford noted that arterial bifurcations were particularly susceptible to PAD along with points of arterial angulation.^19^ While PAD can be limited to a single location, when found at multiple locations, the blood flow to the lower limbs may be severely compromised and claudication or other complications may appear.

### Methodological Issues

#### Estimating Incidence and Prevalence (Descriptive Epidemiology)

##### (i) Diagnosis and misclassification of disease status

In general, there was a lack of uniform case definitions, as well as lack of consensus for non-invasive or invasive diagnostic criteria. This made for inconsistencies across the reported incidence and prevalence data, and frustrated the comparison of studies.

##### (ii) Data sources and biases

Variable Sources were noted, including general practice records, completed questionnaires (some by the subject, others by a trained interviewer), interviews, and annual surveys. Confirmation of deaths was usually by vital statistics/death certificates^2^ and flagging central registries,^20^ though not always. Information bias was also noted e.g. not using a random zero sphygmomanometer,^7^ using incorrect cuff size,^10^ non-standardization of measuring conditions,^21^ measuring ankle blood pressures in the prone position while taking brachial pressures in the supine position,^9^ and variations on standard “Rose questionnaire.”^2^

#### Etiologic Research (Analytic Epidemiology)

##### (i) Confounding

Higher cholesterol levels and smoking rates in men,^21^ and high prevalence of subjects with hyperlipidemia in some cohorts^2^^,^^22^ are some examples of confounding noted in the literature.

##### (ii) Selection biases

Examples included high negative response rates,^7^ patients sampled from ongoing larger cohorts studying different diseases e.g. osteoporosis,^17^ hyperlipidemia,^2^^,^^22^ and Medicare registries.^10^ In addition, detection bias might be very important (i.e. greater likelihood of detection among those with suspected risk factors), possibly more so for women than for men.

## RESULTS

### Diagnosis

Diagnosis of PAD has been based on a variety of modalities, including symptoms and diagnostic testing (both non-invasive and invasive). However, no consensus or clear uniform standards have been defined in the literature as yet.^8^ These diagnostic methodologies will now be reviewed.

#### (i) Symptoms

Intermittent claudication is typically the initial clinical presentation for women with PAD. This calf pain results from atherosclerotic narrowing of the arteries in the femoral and popliteal system. When calf muscles are working, as during walking, oxygen demand may exceed the oxygenated blood supply resulting in the cramping pain of claudication. Finally, when the narrowing becomes severe, pain will be experienced at rest.

Claudication has been assessed using the standardized Rose questionnaire developed at the London School of Hygiene and Tropical Medicine.^23^ This questionnaire has a low sensitivity (about 50%) and a high specificity (80-90%) for detecting PAD in both men and women. Advantages to this questionnaire are that it is repeatable and standardized; however, errors may occur in the completion of the questionnaire, by the patient (if using the self-administered form) or the health care worker (administering the questionnaire). Other disadvantages are that it only detects symptomatic cases, and appears less sensitive in women.^24^^,^^25^

In the largest study of women alone involving 1,601 healthy women aged 65-93 years,^17^ the Rose Questionnaire had a low sensitivity (18%) when compared against the ABI. In the Rotterdam Study of 6,450 participants aged 55 years and older living in the suburb of Ommoord in the Netherlands, the frequency of a positive Rose questionnaire for intermittent claudication was 1.4% for women and 2.1% for men.^26^ Unfortunately, based on the results of non-invasive and invasive tests, claudication by history alone underestimates the true prevalence of PAD.^6^^,^^12^^,^^17^

The Edinburgh Artery Study^20^^,^^27^^,^^28^ was a cohort study of 1,592 (men and women), aged 55-74 years, randomly chosen from 10 general practices, followed to describe the natural history of PAD, with meticulous measurement methods that minimized bias. In this cohort, the prevalence of claudication was 4.5%; major asymptomatic disease causing significant blood flow reduction was found in 8.0%, and a further 16.6% had either abnormal ABI (see below) or reactive hyperemia pressure reduction.^20^ In other words, approximately five times the cases with intermittent claudication by history were detected using other non-invasive tests. They found that one-third of claudicants had normal non-invasive testing suggesting identification of false positives by the Rose questionnaire, and some true claudicants who would be expected to have normal results for any continuous biological variable.^20^ Unfortunately they did not report male and female participants separately. Other false positives occur due to musculoskeletal disease and venous disorders.

False negatives with the Rose questionnaire may occur due to asymptomatic disease, atypical pain, sedentary existence, or other physical conditions limiting activity.^24^^,^^29^ In addition, choice of patients can profoundly bias and hence affect prevalence of claudication, as in the Italian study of elderly institutionalized subjects.^30^ In this study of 124 elderly subjects (97 women and 37 men) in two retirement homes, most reported no symptoms of claudication, leading the authors to speculate this was probably secondary to this populations low physical activity. In addition, 21 subjects (17%) were bedridden on account of hip fractures and were not physically active. Some authors include those patients who report exercise calf pain not relieved within 10 minutes by rest.^2^^,^^31^ This may increase false negatives as patients with spinal stenosis may also experience such pain. A more recent modified version of the Rose questionnaire has improved sensitivity in both sexes yet still far below other non-invasive instruments.^32^

#### (ii) Diagnostic Testing

##### Ankle-Brachial Index (ABI)

Another test involves having the patient lie supine and taking a blood pressure at the ankle using an ultrasound probe overlying the posterior tibial and the dorsalis pedis artery, as well as a routine blood pressure in the brachial artery in the arm, and then taking the ratio of this measurement to calculate the ankle-brachial index (ABI) i.e. the ratio of the lower extremity (ankle) systolic blood pressure to upper extremity (brachial) systolic blood pressure. The ABI is also referred to as an ankle-arm ratio or index, the ankle-brachial pressure index, or the Winsor Index.^7^^,^^24^^,^^29^^,^^33^^,^^34^

The normal ABI is 1.0 - 1.5 (bilaterally), since the pressure is higher in the ankle than in the arm.^10^ An ABI of < 1.0 but > 0.90 is classified as borderline or mildly abnormal.^35^ An ABI < 0.90 has a greater than 90% sensitivity and a 95% specificity for detecting angiogram-positive PAD.^24^ An ABI of 0.5 to 0.84 suggests a degree of arterial obstruction often associated with claudication, and an ABI < 0.5 represents advanced ischemia and is associated with a poor prognosis in women.^15^ On the other hand, an ABI > 1.5 suggests arterial rigidity preventing arterial occlusion.^10^ For example, diabetic patients may have a falsely elevated ABI due to calcific vessels that do not compress.

In the PARTNERS Program (Peripheral arterial disease Awareness, Risk and Treatment: NEW Resources for Survival), patients with PAD had a mean ABI of 0.78, compared to 1.09 for those without PAD.^6^

Several studies noted that the average resting ABI was lower in women than in men.^20^^,^^36^ In the Rotterdam study,^8^ for example, the mean ABI in women was 1.03 (standard deviation 0.23) compared with men 1.08 (standard deviation 0.24). ABI detects at least 4-5 fold more PAD in women than intermittent claudication history.^17^ In addition, women appear in some studies to have more asymptomatic disease than men.^7^

The main advantage to the ABI test is that it is reproducible, and has a high sensitivity and specificity.^6^ While the ABI appears sensitive when compared to angiogram, this comparison stems from studies that were hospital based, using selected hospital patients and controls judged to be healthy because they were young or had no symptoms or signs. These hospital cases and controls are by definition ill and therefore differ from the general population in various ways that may be associated with illness or hospitalization, such as increased rates of smoking, oral contraceptives use, and alcohol consumption, all of which may lead to various biases in these studies.^37^ Disadvantages are that one requires a doppler probe, and some patients may not be able to tolerate the test.^17^

Variations on performing ABI across studies was common, including which arm(s) and leg(s) & which systolic blood pressure reading was analyzed (highest, lowest, or average). For instance, the Pittsburgh mortality study took pressures in both arms and legs, then took the lower of the ankle readings over the higher of the brachial measures, which would underestimate the ABI.^1^

No studies controlled for ambient temperature; blood pressures measured in summer have been noted to be lower than in winter.^38^ Also, most studies contained digit preference bias in determining blood pressure; only the Edinburgh and the Jerusalem groups utilized a Hawksley random zero sphygmomanometer.^22^^,^^24^

##### Ultrasound

Color and pulsed doppler imaging uses a probe to measure blood flow within an artery. An abnormal doppler waveform is detected distal to an arterial stenosis, helping to diagnose location, severity and frequency of disease.^39^ Additionally, ultrasound contrast agents, three-dimensional imaging, and B-flow imaging each seem to have improved the ability of ultrasound to assess PAD in women.^40^

##### Magnetic resonance angiography

Two or three-dimensional mode magnetic resonance angiography are becoming an important noninvasive method for assessment of PAD, and are probably the way of the future.^41^^,^^42^ Magnetic resonance angiography is sensitive (90-94%), specific (90-94%), reproducible, and assists in interventional and surgical planing. Its downside includes need for expensive equipment, as well as time consuming studies.^42^^,^^43^

A recent multicenter trial^44^ compared contrast angiography to magnetic resonance angiography. Both were equivalent in diagnostic accuracy. Magnetic resonance angiography, however, provided more information and helped improve surgical planning for approximately one sixth of the patients scheduled for vascular surgery. This study did not analyze the 97 men and 58 women separately.

##### Invasive Testing

Peripheral contrast arteriography is currently the gold standard for evaluating PAD. It reveals arterial anatomic detail, location and morphology of atherosclerotic disease, and allows for estimation of the degree of narrowing from plaque buildup. One must remember that what we are measuring with arteriography is a luminogram. Endothelial or medial wall thickness, which is believed to be the early changes in the atherosclerotic process, may be present prior to any change in lumen size.^45^ Thus, it is important to point out that none of these invasive or non-invasive instruments measures endothelial dysfunction, which is believed to underlie and precede the pathological atherosclerotic process.^46^

### Epidemiology

#### Incidence

The Framingham Study^16^^,^^47^^,^^48^ was a cohort study of 5,209 subjects (men and women) aged 29-62 years initially recruited in 1949. Based on development of intermittent claudication over 20 years, Framingham gave a biennial PAD incidence rate of 3.5/1000 for women and 7.1/1000 for men.^16^

#### Prevalence

Prevalence of PAD determined from intermittent claudication history in men and women is presented (Table 1); several studies unfortunately did not provide gender specific prevalence values for intermittent claudication. PAD prevalence in women by intermittent claudication was low in general (ranged 1.0 to 12.7%).

**Table 1.** Prevalence of peripheral arterial disease (PAD) by intermittent claudication (IC).

Location, Study Type, Author	Year	n	Ages	Prevalence (%)	

				Men	Women
PARTNERS^a^, 320 primary care practices in US, Hirsch et al.^6^	2001	6,417	≥ 70 or 50- 69 (smokers and/or diabetic)	12.7^b,c^	12.7^b,c^

Rotterdam, Cross-Sectional, Meijer et al.^8^	1998	7,715	≥ 55	2.2	1.2

ARIC^d^ study, four US communities Zheng et al.^9^	1997	15,792	45-64	1.0	1.0

Limburg, Cross-Sectional, Stoffers et al.^7^	1996	3,171	45-54	0.5	0.3
			55-64	0.9	2.2
			65-74	2.4	3.7

Cardiovascular Health Study, four US communities, Newman et al.^10^	1993	5,084	≥ 65	2.0^b^	2.0^b^

Pittsburgh, Cross-Sectional, Vogt et al.^17^	1993	1,491	65-93	-	7.4

SHEP^c^ study, sub-group, Newman et al.^70^	1993	1,775	≥ 65	6.4^c^	6.4^c^

California, Cross-Sectional, Criqui et al.^31^	1992	613	38-82	2.2	1.7

Edinburgh, Cohort Study, Fowkes et al.^20^	1991	1,592	55-74	4.5^c^	4.5^c^

Jerusalem, Cross-Sectional, Gofin et al.^22^	1987	1,592	35-64	1.3	1.8

Denmark, Cross-Sectional, Schroll & Munck^25^	1981	661	60	5.8	1.3

^a^ Peripheral arterial disease Awareness, Risk and Treatment: NEw Resources for Survival Program

^b^ Prevalence in the total population; no separate estimates for gender were reported.

^c^ The PARTNERS program gave data for “chart history of claudication;” Rose claudication numbers were not given, though they do state “charted claudication was much more common than classic (questionnaire-based) Rose claudication.”^71^

^d^ Atherosclerosis Risk In Communities Study

^e^ Systolic Hypertension in the Elderly Program

PAD may be better determined by ABI < 0.90. In all studies including women, prevalence rates vary from 3.0 to 29.0% depending on the age group, ABI cut point, and risk factor profiles of the cohort studied.^6^^,^^9^^,^^20^^,^^29^^,^^31^ A summary of PAD prevalence using this cutoff value unless shown otherwise is presented (Table 2). Most studies confirmed that ABI detects significantly more PAD in women than history of intermittent claudication.

**Table 2.** Prevalence of peripheral arterial disease (PAD) by Ankle-Brachial Index (ABI).

Location, Study Type, Authors	Year	n	Ages	ABI cutoff	Prevalence (%)	

					Men	Women
PARTNERS^a^ 320 primary care practices in US, Hirsch et al.^6^	2001	6,417	≥ 70 or 50-69 (smoker s and/or diabetic)	≤ 0.90^b^	29.1^c^	29.1^c^

Rotterdam, Cross-Sectional, Meijer et al.^8^	1998	7,715	≥ 55	< 0.90	16.9	20.5

ARIC^d^ study, four US communities Zheng et al.^9^	1997	15,792	45-64	< 0.90	3.0	3.0

Limburg, Cross-Sectional, Stoffers et al.^7^	1996	3,171	45-54	< 0.95	1.6	3.1
			55-64	< 0.95	7.7	6.8
			65-74	< 0.95	16.4	11.2

Cardiovascular Health Study, four US communities, Newman et al.^10^	1993	5,084	≥ 65	< 0.90	13.8	11.4

Pittsburgh, Cross-Sectional, Vogt et al.^17^	1993	1,492	65-93	≤ 0.90	-	5.5

SHEP^c^ study, sub-group, Newman et al.^70^	1993	1,775	≥ 65	< 0.90	26.7^c^	26.7^c^

California, Cross-Sectional, Criqui et al.^31^	1992	613	38-82	tests^f^	11.7	11.7

Edinburgh, Cohort Study, Fowkes et al.^20^	1991	1,592	55-74	≤ 0.90	18.3^c^	18.3^c^

Jerusalem, Cross-Sectional, Gofin et al.^22^	1987	1,592	35-64	< 0.90	4.2	5.4

Denmark, Cross-Sectional, Schroll & Munck^25^	1981	661	60	< 0.90	16	13

^a^ The PARTNERS program (Peripheral arterial disease Awareness, Risk and Treatment: NEw Resources for Survival)

^b^ The PARTNERS program defined PAD as either an ABI ≤ 0.90, a previous history of PAD, or prior limb revascularization.

^c^ Prevalence in the total population; no separate estimates for gender were reported.

^d^ Atherosclerosis Risk In Communities Study

^e^ Systolic Hypertension in the Elderly Program

^f^ Criqui et al.^31^ used a different approach to assess the prevalence of PAD; the standard ABI was not used, but rather 4 different noninvasive tests (segmental blood pressure, flow velocity by doppler ultrasound, post-occlusive reactive hyperemia, and pulse reappearance half-time) were used to diagnose PAD.

The Pittsburgh Study of Healthy Elderly Women was a cross-sectional study of 1,601 healthy women, aged 65-93 years (mean 71 years) taken from patients involved in the Multicenter Study of Osteoporotic Fractures.^17^ They excluded 104 women because they refused ankle blood pressures, hence this study was subject to selection bias. Both the Rose questionnaire and ABI < 0.90 were utilized. Using the later instrument, they noted that PAD prevalence increased with age (2.9% at 65-69 years increasing to 15.5% at 80 and older years). Also, only a fifth of women with PAD by ABI criteria had symptoms.

The Limburg Study was an observational study of 3,171 patients from 18 general practices, men and women, aged 45-74 years, derived randomly from a pool of 23,004 subjects who responded to a postal questionnaire.^7^ Diagnostic instruments included an intermittent claudication questionnaire, and ABI (positive if < 0.95) checked on two separate measurements. They found that of all PAD cases, only 22% were symptomatic; and of these, only 25% were females. General practitioners diagnosed PAD most often among men, especially in age range 55-74 years. The prevalence of PAD by ABI was 6.5% among women, and 7.2% among men. The proportion of symptomatic cases correlated positively with age, male gender, and lower ABI. Thus, in this study, females were less likely than men to be symptomatic (11% vs. 32%).

Case definition affects results with higher values of prevalence obtained if “small vessel” PAD is included. If one includes major and minor asymptomatic cases with symptomatic cases, as reported by Fowkes et al.,^20^ prevalence values of 29% (women) and 27% (men) are indicated, assuming there is no overlap in the minor and major asymptomatic cases. The PAD prevalence record must go to Paris et al.^49^ who report a value of 88% for New York nursing home residents; these 60 individuals (80% females) were at least 80 years of age and were identified as PAD cases using ABI < 0.95 criteria.

#### Comorbidity

Among women, those with PAD have higher vascular and non-vascular morbidity. In the Pittsburgh rural community study of 1,492 women 65 years of age or older, 82 (5.5%) were found to have a ABI < 0.90; when compared to the remaining women (after adjustment for age, smoking, and other risk factors), these women had a relative risk four years later for heart disease of 3.7, for cardiovascular diseases 4.0, and for cancer 3.3.^50^ In addition, ABI is closely associated with lower-extremity function.^51^

In the Rotterdam study, women with PAD (ABI < 0.90) frequently had a history of cardiovascular disease: 9.2% angina pectoris, 15.0% myocardial infarction, and 8.4% stroke.^8^ The percentages for angina and stroke were similar in men; however, men with PAD were more likely to have had a myocardial infarction (29.9%).

In Edinburgh, of those with major asymptomatic PAD, 54% had evidence of coronary artery disease, 1.6 times that in the normal population.^20^ It is possible that recall bias was artificially inflating these numbers. The authors did their best to minimize other bias, for example, having the electrocardiograms coded independently by two trained staff using the ‘Minnesota Code.’ Unfortunately due to few events, a gender analysis was not reported.

The PARTNERS program enrolled 6,979 patients in a large number of primary care practices using simple screening criteria - age over 70 or age over 50 with a history of diabetes or smoking - and is shedding light on PAD and its association with other cardiovascular disease (CVD). In PARTNERS, CVD was defined as history of angina, myocardial infarction, coronary artery bypass grafting, percutaneous transluminal coronary angioplasty, abdominal aortic aneurysm, transient ischemic attack, or stroke. Of the patients enrolled, 16% had PAD and CVD, 13% had PAD but no CVD, 24% had no PAD but did have CVD, and 47% had evidence of neither.^6^ Of those with PAD (n = 1865), evidence of cardiovascular disease was noted in 56%.^6^ Even though there were approximately equal proportions of women (52%) and men (48%) overall, women made up 61% of the group diagnosed with PAD only; however, they made up a smaller amount (44%) of the group who had both PAD and CVD together. This suggests that women with PAD in this population had less cardiovascular diseases than men with PAD.

Women tend to have a poorer prognosis following vascular surgery;^52^^,^^53^ though not always.^54^ Suggested causes why women may have less success with surgery include delayed diagnosis, decreased referrals, increased comorbidity, more postoperative complications, and possibly the effect of their smaller arteries.^11^^,^^53^^,^^54^

#### Mortality

PAD is considered a manifestation of generalized atherosclerosis, and thus is associated with increased CVD and poor cardiovascular prognosis.^8^ Thus it is not surprising that the cause of death in approximately two-thirds of women with PAD is myocardial ischemia, followed by cerebrovascular disease, ruptured aneurysms, and visceral infarctions.^15^

Vogt et al’s study of 1,492 Pittsburgh women in rural communities found that those with PAD, when compared to the remaining women, had an adjusted relative risk for all-cause mortality four years later of 3.1.^50^ In the Systolic Hypertension in the Elderly Program (SHEP) substudy involving 1,537 participants (868 women) with PAD (ABI < 0.90) who were followed for four years, the age adjusted relative risk for morality was 2.8 in women (compared with 3.0 in men). These results persisted after adjustment for cardiovascular risk factors.^55^

Another study identified and studied a group of 565 men and women aged 38 to 82 years and followed them for 10 years.^2^ Their cohort consisted of predominantly white upper-middle-class community members in Southern California who were previously members of the Lipid Research Clinics Protocol (this method of sampling would be expected to introduce selection and recall bias, and possible confounding). Of a total of 4,535 subjects who attended the lipid research clinic for their first visit, only 1,435 subjects were invited to return for a second visit, half of whom had hyperlipidemia. They sampled 624 eligible subjects; 9.5% were excluded (missing data) leaving 565 subjects. While other researchers have used the Rose questionnaire, these authors utilize the Rose questionnaire and also included “possible” claudication defined as “calf pain present on exercise but not at rest that otherwise did not fully meet the Rose criteria,” which increased cases, yet validation was not discussed.^2^^,^^31^ After multivariate adjustment for age, sex, and cardiac risk factors, Criqui et al.^2^ found an increased risk of dying among persons with large vessel PAD compared to normals (relative risk 3.1). In addition, they found a gradient of mortality increasing from asymptomatic to symptomatic to severe symptomatic PAD. Because the authors decided that the association of large vessel PAD with mortality was similar in men and women, PAD mortality data by gender was not provided.

ABI as an independent predictor of mortality in women was confirmed by yet another study out of Pittsburgh.^1^ This retrospective study analyzed 1,038 patients that had undergone noninvasive testing prior to 1985 in a university affiliated community hospital. Approximately 28% of patients were excluded for reasons including missing data, non-residents of Pennsylvania, or because they had undergone previous vascular surgery; 744 patients (54% females) remained. Sources of information for death ascertainment included the Pennsylvania index of deaths of all residents, and the corresponding International Classification of Diseases coded death certificate. Such state sources may have underestimated actual mortality. Using an ABI < 0.85 as their diagnostic criteria for PAD, the relative risk for total mortality was 2.4. There was a gradient effect with increasing mortality with lower ABI, with an ABI < 0.40 giving a total mortality relative risk of 4.5. Risks remained significant after adjustment for age and multiple risk factors. There was no significant difference between mortality for men and women in this study.^1^

Mortality for women with PAD thus appears to be similar to that for men with PAD, with a relative risk of dying of two to three fold across the studies reviewed.

### Gender Differences

#### Symptomatic versus Asymptomatic

The incidence or prevalence of PAD has been difficult to determine because of the lack of reliable data on asymptomatic cases. Previously, many authors focussed on symptomatic cases - mainly presenting with intermittent claudication - which significantly underestimates true PAD cases. Stoffers et al.^7^ with a sample of 3,171 (women and men) state that 22% of all PAD cases were symptomatic, of which 25% were female; this means that 78% are asymptomatic, 55% of whom are females. Thus, women in this study had more asymptomatic PAD than men (89% vs. 68%). Also, as the ABI decreased, the percentage of symptomatic patients increased, again women less symptomatic than men. Of interest, the symptomatic and asymptomatic groups did not differ significantly in proportion having coronary artery disease or cerebrovascular disease. By using a higher cutoff value (ABI < 0.95), this study may have overestimated asymptomatic cases, while diminishing their specificity.

For an ABI ≤ 0.90, Vogt et al.^17^ results allowed the authors to calculate that for this group of elderly women, 82% were asymptomatic (no intermittent claudication). Recent literature indicates asymptomatic cases are higher than symptomatic cases of PAD in both sexes, and in some studies, more so in women. For instance, in the Rotterdam study, women with PAD (ABI < 0.90) complained of symptoms of intermittent claudication less often than men, 4.9% versus 8.7%, respectively.^8^

Women with asymptomatic PAD have reported more difficulty with activities of daily living, are less active, have lower physical endurance, diminished leg strength, and have a slower cadence, all of which may mask symptoms of intermittent claudication.^29^^,^^56^^,^^57^ In addition, women may present more frequently with atypical symptoms of PAD, as has been noted in other ischemic diseases such as coronary heart disease.^8^^,^^58^

Thus, the true prevalence of PAD in women is expected to be much higher than originally thought, at least three to five fold the prevalence of intermittent claudication.^20^

#### Is there a gender difference in PAD prevalence?

The earlier literature contends that PAD prevalence is higher among men than women for most age periods, with a catch-up phenomenon occurring in postmenopausal women such that the gender difference diminishes and prevalence in women trends towards men. This conclusion was based on the comparison of claudicants to non-claudicants. Recent literature, based on more sensitive and specific non-invasive measures of PAD, such as ABI, does not support a gender difference.^10^^,^^20^ Interestingly, in the early results of the PARTNERS program, the ABI was lower in women (Dr Alan T. Hirsch MD, University of Minnesota Medical School, “personal communication,” 2001). This finding, which has been noted in other studies,^8^^,^^20^^,^^36^ may be a true gender effect. It may also in part explained by the fact that women’s mean height is less than men’s, ABI having been noted to correlate with height.^20^ Yet, even after additional adjustment for height, the Edinburgh study still found a significant though small sex difference with mean ABI still lower in women.^20^ Thus, whether there is a true difference in prevalence of asymptomatic PAD between men and women is difficult to determine because of possible lower mean ABI in women. Yet, even so, the absolute difference in ABI between men and women is very small, for instance 0.05 in the Rotterdam Study.^8^

#### Hormonal factors

The literature suggests a possible association between hormonal factors and the development of PAD in women along several lines.

First, the incidence of intermittent claudication is less for premenopausal women but approaches that of men when these women reach the sixth and seventh decades, and has almost caught up with men by age 80.^59^ In Framingham, the incidence of intermittent claudication in women more than doubled from 1.7% at ages 45-54 to 4.7% at ages 55-64.^16^ However, more recent cohorts which defined prevalence based on ABI, a more sensitive instrument for detecting PAD, showed little if any sex predilection. In addition, in the Limburg and Jerusalem studies, women in the younger age ranges, some even premenopausal, had a higher prevalence of PAD by ABI than men.^7^^,^^22^ Thus, in these studies mainly containing post-menopausal women, prevalence of PAD does not appear to be significantly different, suggesting no natural hormonal protective effect.

Second, both total and low density lipoprotein-cholesterol levels increase during the postmenopausal years, while high density lipoprotein-cholesterol remains unchanged. In the Cardiovascular Health Study, these lipid components significantly increased risk of developing PAD in women but not in men.^10^ Thus, a women’s risk may accelerate following the menopause.

Third, in the Heart and Estrogen/progestin Replacement Study, a randomized clinical trial of secondary prevention of coronary heart disease with estrogen and progestin, there was a trend in the estrogen plus progestin group toward less cases of PAD (94 versus 108 cases) with a Relative Hazard of 0.87 (95% confidence interval 0.66-1.15, p = 0.34).^60^ Evidence to the contrary comes from a nested case-control study as a part of the Edinburgh cohort that analyzed mean levels of estradiol, sex hormone binding globulin, and free testosterone, and showed no differences between cases and controls in either women or men.^28^

Fourth, in a population-based study of 2,196 naturally menopausal women aged 55 to 80 years, and using the ABI < 0.90 cutoff, hormone replacement therapy for one year or longer was associated with a 52% decreased risk of PAD (odds ratio, 0.48, 95% confidence interval, 0.24-0.85), while no association was found for therapy duration shorter than one year (odds ratio, 0.97, 95% confidence interval, 0.58-1.63) after adjustment for age, smoking, and socioeconomic status. Additional adjustment for body mass index, age at menopause, total cholesterol and high-density lipoprotein cholesterol, alcohol intake, and frequency of visits to health care facilities did not change the results.^61^

Fifth, in the PARTNERS program, by definition, all the women in the study population were aged 50 years or over; use of postmenopausal hormone replacement therapy was low overall.^6^ Yet, hormone replacement therapy use was higher in the group with no PAD or CVD compared to the other groups manifesting atherosclerosis i.e. PAD, CVD, or both (p<0.01).

Sixth, in postmenopausal women, estrogen has been shown to improve endothelial function, and may thus have atheroprotective effects and slow the development of PAD.^62^^,^^63^

While premenopausal naturally produced estrogen does not appear to have a significant protective effect against PAD, there is a suggestion that post menopausal hormone replacement therapy for at least a year may decrease a women’s risk of developing PAD.

### Risk Factors

The Framingham study was the first to catalogue risk factors using history of claudication, and found risk factors for PAD overlapped those for coronary heart disease.^16^^,^^47^ Most authors present risk factors for PAD that are similar to those for coronary heart disease. Of note, in some studies there was a gender difference in both the significance of some risk factors as well as the strength of the association. Table 3 summarizes the main associations found with risk factors and PAD in women.

**Table 3.** Risk factors and their associations for peripheral arterial disease (PAD) in women and men.

Risk Factor	Women	Men
Cigarette smoking^13^^,^^17^^,^^22^^,^^72^^,^^73^	OR 2.7 - 6.4	OR 1.7 - 2.6

Hypertension^13^^,^^74^	OR 2.2 - 2.7	OR 2.2

Systolic blood pressure^17^^,^^75^	OR 1.4 (per 10 mm Hg)	Not associated (there may be an association with diastolic blood pressure)

Hyperlipidemia^10^^,^^13^^,^^74^^,^^76^^-^^79^	Total cholesterol	
	Low-density lipoprotein cholesterol OR 1.02	Low-density lipoprotein cholesterol OR 1.02
	High-density lipoprotein cholesterol OR 0.90-0.97	High-density lipoprotein cholesterol OR 0.90-0.95
	Triglyceride	Triglyceride

Diabetes mellitus^10^^,^^13^^,^^80^^,^^81^	OR 2.3 - 3.6	OR 1.6 - 6.0

Homocysteine^82^^-^^86^	OR 3.0 - 6.0	OR 1.0 - 6.0

Fibrinogen^a ^^74^^,^^87^^-^^89^	RR 1.0-1.3	RR 1.0-1.3

C-reactive protein^86^^,^^90^	Positively associated (risk ratio not available)	RR 1.3-2.8

^a^ no separate estimates for gender were reported.

OR: odds ratio

RR: relative risk

## DISCUSSION

PAD goes largely undiagnosed in women. History of calf pain is inefficient for diagnosis, identifying less than one-third of prevalent cases. During the physical examination, checking peripheral pulses and performing an ABI could potentially triple the identification of PAD in women. Importantly, women are less likely to have symptoms such as claudication, and thus more likely to have asymptomatic disease. Correctly identifying women with PAD is extremely important as this group has an accelerated morbidity and mortality when compared with controls. Key aspects of PAD specific to women are summarized (Table 4).

**Table 4.** Specific features of peripheral arterial disease (PAD) in women.

Aspect	Key points
Diagnosis	Women less likely to present with intermittent claudication
	Most women (50-90%) with PAD have asymptomatic disease
	Mean ankle-brachial index (ABI) lower in women
	Prevalence of PAD by ABI is about equal in women and men

Morbidity	Women with PAD have up to four times the risk of cardiovascular disease (CVD)
	Women do worse following vascular surgery for PAD

Mortality	Women with PAD have a risk of death about three fold those without PAD

Risk Factors	Total cholesterol, low-density cholesterol, diabetes, and smoking may increase risk for PAD in women more than in men
	Elevated systolic blood pressure increases a women’s risk of PAD
	Other major risk factors afford equal risk in women and men.
	Postmenopausal hormone replacement therapy for a year or more may decrease risk of subsequent PAD

The question remains “Has the instrument itself altered the male : female differences in PAD prevalence?” In epidemiological studies in the 1950’s and 60’s, premenopausal women appeared to be less affected than men, but their prevalence approached men in the sixth and seven decades. These studies based the diagnosis of PAD on an intermittent claudication questionnaire which, in retrospect, had a low sensitivity and underestimated the true prevalence of PAD. Based on these earlier studies, there also appeared a clear gender difference with fewer cases occurring in women. This hypothesis also conveniently paralleled prevalence in coronary artery disease, which shows a similar premenopausal nadir and a postmenopausal rise. More recent studies have not detected a gender difference in prevalence of PAD. Why? The case definition in these studies is different.

More recent and well constructed cohorts, using more sensitive instruments to detect PAD, have concluded that men and women are essentially no different with regard to the prevalence of PAD. In addition, women may make up as much if not more of the asymptomatic group, which importantly confers significant morbidity and mortality on them.

Smoking and cholesterol appear to induce an even higher risk for PAD in women. Because of the strong overlap of these and other risk factors, and the frequent association of PAD with cardiovascular and cerebrovascular disorders in women, these risk factors should be treated aggressively.^64^ In particular, with rising rates of smoking in women, risk factor targeting and modification is paramount.

The jury is still out on hormone replacement therapy and its impact on PAD in women, with several studies suggesting it may be helpful if used for a year or more. However, given recent trial results with other cardiovascular endpoints, hormone replacement at this time seems unwise. Clearly more research is needed in effects of risk factor modification on subsequent development of PAD in women. This could include estrogen and progesterone replacement, lipid lowering, antihypertensive medications, control of diabetes, and treating elevated homocysteine levels. Ongoing randomized trials include the Women’s Health Initiative and the WELL-HEART trials, which are looking at primary and secondary coronary prevention in addition to PAD in women.^65^

Should ABI be part of the routine physical in elderly women? Several authors ^7^^,^^24^ suggest that given the low detection of PAD in women due to lack of symptoms, measurement of the ABI may be useful in two respects. Firstly, identification of asymptomatic women with PAD; and secondly, identification of these women at risk for coronary disease and stroke. In identifying and flagging such women as high risk patients, the physician can target both primary and secondary preventive efforts for PAD, coronary artery disease, and cerebrovascular disease. If asymptomatic PAD in women is detected early, and ongoing randomized studies prove benefit from earlier treatment and aggressive risk factor modification including exercise, we may be able to stem the loss of mobility, as well as the high morbidity and mortality of this condition.

We further recommend the following:

(i) Studies to determine which cut-off point should be used with respect to the ABI to diagnose PAD in women. This is important as it appears that the mean ABI is lower in women. This would require comparing different ABI cut points against various gold standards such as angiography, ultrasonography, and magnetic resonance angiography. Perhaps a combination of instruments may prove the best strategy in diagnosing PAD in women as each has their advantages and disadvantages.

(ii) Randomized controlled trials to define whether, by screening and detecting female PAD cases earlier (mainly asymptomatic cases), we can decrease subsequent morbidity and mortality by aggressive risk factor modification. Of note, the UK Prospective Diabetes Study group 38 trial found a trend toward decreased rate of PAD, fewer amputations, and lower mortality from PAD in the intensively treated diabetics, as well as those whose systolic blood pressure was tightly controlled.^66^

With respect to the future, clearly more knowledge of the biochemistry and molecular biology underlying atherosclerosis, in addition to angiogenesis-related growth and transcription factors, will influence the prevention and correction strategies towards PAD in both women and men.^46^^,^^67^^-^^69^ What is currently clear is that PAD is common in women, and recognizing this entity may represent another opportunity for beneficial risk factor modification in this population.
